# Supramolecular Polycaprolactone-Based Polyurethanes with Thermally Activated Shape-Memory Behavior

**DOI:** 10.3390/polym14173447

**Published:** 2022-08-24

**Authors:** Fabio Muscas, Valentina Sessini, Laura Peponi, Antonio Julio López, Alejandro Ureña, Rodrigo Navarro, Ángel Marcos-Fernández

**Affiliations:** 1 Departamento de Matemática Aplicada, Ciencia e Ingeniería de Materiales y Tecnología Electrónica, ESCET, Universidad Rey Juan Carlos, 28933 Madrid, Spain; 2 Instituto de Ciencia y Tecnología de Polímeros (ICTP-CSIC), Calle Juan de la Cierva 3, 28006 Madrid, Spain

**Keywords:** 2-ureido-4-[1H]-pyrimidinone, quadruple hydrogen bonds, PU, stimuli responsive

## Abstract

In this work, using supramolecular polyurethanes theories, two polycaprolactone-based polyurethanes with 2-ureido-4-[1H]-pyrimidinone (UPy) motifs capable of forming quadruple hydrogen bonds were synthetized and characterized, focusing our attention on their capability to show thermally activated shape-memory response. In particular, ^1^H NMR analyses confirmed the chemical structure of the supramolecular polyurethanes, while DSC showed their totally amorphous morphology. DMTA in tensile mode was used to study their thermally activated shape-memory properties. In our case, the UPy units are the switching domains while the network formed by the segregated hard segments is the permanent domain obtained materials with excellent shape-memory response at both 100 and 85 °C. These materials are promising for multi-responsive materials where bio-based and potentially recyclable polymers with excellent shape-memory properties are needed.

## 1. Introduction

In recent decades, shape-memory polymers (SMPs) have gained great interest as a new class of stimuli-responsive materials for potential application in the biomedical field. They present remarkable properties, such as easy processability, low weight and relatively low cost, with the added values of high elastic deformation and good capability to fix a temporary shape and to recover its initial shape within the application of a large range of stimuli [1,2,3,4]. In particular, thermally activated shape-memory polymers can recover their permanent shape upon exposure to temperature as external stimulus [5]. In order to show shape memory, a reversible switching network as well as a stable permanent network are required [6]. The permanent domains which can be formed by molecule entanglement, crystalline phase, chemical cross-linking or interpenetrated network are able to memorize the original shape while the switching network is responsible for fixing the temporary shape and can be related to crystallization/melting transition, glass/rubber transition, reversible cross-linking and supramolecular association/dissociation [7]. Recently, the use of reversible cross-linking bonds and supramolecular polymer networks as a switching network has been proposed due to their reversible and stimuli-responsive nature [8].

In particular, supramolecular polymers consist of relatively low-molecular-weight species that are able to assemble, triggered by temperature, into higher ordered structures. Thanks to specific designed motifs, they can form reversible non-covalent bonds that control the mechanical properties of the final material [9,10]. These molecules’ self-associations can lead to the formation of highly complex non-covalent interactions, including metal coordination, electrostatic interactions, Van der Waals forces, π-π interactions and hydrogen bonds [11]. Therefore, hydrogen bonds play an important role in determining the three-dimensional chemical structure of these systems. Thus, an increase in the number of hydrogen bonds within the molecular structure would enhance the number of non-covalent interactions leading to the design of new multifunctional polymers [12]. In contrast to covalent bonds, hydrogen bonds are reversible, and their strength depends on temperature as well as their concentration in the chemical environment, such as in a solvent. The 2-ureido-4-[1H]-pyrimidinone (UPy) moiety is known to dimerize via strong quadruple hydrogen bonding. These units with extremely high dimerization constants (*K_dim_* = 6 · 107 M^−1^ in CHCl_3_) [13,14] have received significant attention for the construction of novel supramolecular architectures. In such systems, different properties, such as mechanical properties and viscosity of the supramolecular polymers, can change by increasing temperature, showing thermo-responsive behavior based on reversible hydrogen bond interactions [15]. Thus, UPy groups are ideal for designing thermally responsive supramolecular materials with a wide range of interesting properties, such as degradability, recyclability, shape-memory and self-healing properties [12], providing applications such as adhesives, healable coatings, shape-memory materials and impact-resistant structures (e.g., protection for mobile electronics) [16].

As reported in the scientific literature, on the one hand, UPys are used to obtain SMPs with excellent shape-memory properties [17,18] using the UPy dynamic network as the switching network [19,20] and, on the other hand, it was demonstrated that the UPy network can also be used to memorize the original shape and thus be used as a permanent network [21,22,23,24,25,26]. Anthamatten et al. reported the use of UPy groups as a thermally responsive switching segment in cross-linked SMPs [18]. The use of a low concentration (2 mol%) of UPy in the covalent cross-linked poly(butylacrylate)-based random copolymers leads to a soft, elastomeric material with a broad transition centered around 50 °C attributed to the UPy dissociation. Ware et al. reported a one-pot method to synthetize chemically cross-linked butyl acrylate/methacrylate random copolymer networks containing UPys with triple shape-memory behavior. In this case, triple shape-memory properties arise from the combination of the glass transition temperature (*T_g_*) and the dissociation temperature of UPy [20]. Song et al. reported that by introducing UPy into polyurethane backbone, thermally and pH-activated shape-memory properties as well as heat-induced healing can be achieved. In these systems, UPy dimers were used as switch segment to control the temporary shape [27]. More recently, Song et al. synthetized thermally and photo-thermally responsive hydrogels with high UPy contents as a switching phase, showing excellent shape-memory ability [28].

In this work, a one-pot method for the synthesis of new shape-memory supramolecular polycaprolactone (PCL)-based polyurethanes containing UPy motifs in the backbone is reported. In particular, two shape-memory PCL-based polyurethanes were synthetized by using UPy motifs as a switching segment with soft segments (SS) of different lengths and the same percentage of hard segment, high enough to ensure the formation of a stable permanent domain. Their thermal, thermo-dynamic mechanical and mechanical properties were studied, as well as their thermally activated shape-memory response.

## 2. Materials and Methods

### 2.1. Chemicals and Reagents

Commercial polycaprolactone (PCL) diol Capa^®^ 2203A (Mn = 2054 g·mol^−1^) was kindly supplied from Perstorp (UK). PCL diol 531 (Mn = 531 g·mol^−1^) was synthetized as reported elsewhere [29,30] by using diethylene glycol as the initiator for the ring opening polymerization of *ε*-caprolactone. The exact molecular weight of PCLs was calculated from proton NMR spectra as already reported [31]. PCLs were dried in vacuum at 100 °C for 3 h and stored in vacuum before use. Stannous octoate (SnOct_2_), guanidine carbonate salt, hexamethylene diisocyanate (HDI), triethylamine (TEA) and hydrochloric acid (HCl) (1M) were purchased from Sigma-Aldrich Química (Madrid, Spain). HDI and TEA were distilled under reduced pressure before use. 2-Acetylbutyrolactone and absolute ethanol (analytical grade) were supplied by Carbosynth (UK) and Scharlau (Barcelona, Spain), respectively, and used as received. *N*,*N*-dimethylacetamide (DMAc) (synthesis grade) was supplied by Scharlau (Barcelona, Spain) and purified by distillation from isocyanates (commercial polymeric MDI) under reduced pressure before use [32].

### 2.2. Preparation of 2-Amino-5-(2-hydroxyethyl)-6-methylpyrimidin-4-ol (AMINE-OL)

**Scheme 1:**
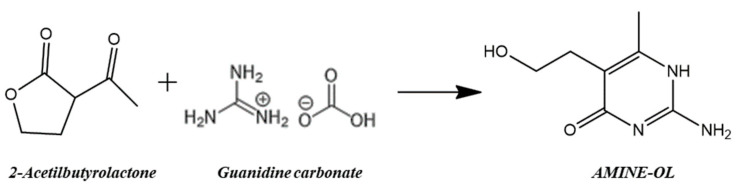
Synthesis of 2-Amino-5-(2-hydroxyethyl)-6-methylpyrimidin-4-ol (AMINE-OL).

2-Acetylbutyrolactone (22 mL, 0.243 mol) and guanidine carbonate (15 g, 0.126 mol) were refluxed in absolute ethanol (100 mL) in the presence of triethylamine (55 mL) in order to obtain 2-Amino-5-(2-hydroxyethyl)-6-methylpyrimidin-4-ol (AMINE-OL) (Scheme 1).

The solution became yellow and turbid. The mixture was stirred at reflux overnight at 80 °C and then the solid was filtered, washed with ethanol and finally suspended in water. The pH was adjusted to a value of 6–7 with a HCl solution. The neutral suspension was filtered and the solid washed with ethanol [33]. The obtained white solid was dried under vacuum for 24 h at room temperature (yield 65%).

### 2.3. Preparation of the Polyurethane Based on PCL531 with a 45% Hard Segment (PUPCL531)

PCL-based polyurethane synthesized using PCL diol 531 and 45% of hard segment, namely, PUPCL531, was prepared by a one-step reaction. The synthetized AMINE-OL (2.6404 g, 15.60 mmol), HDI (5.9547 g, 35.40 mmol) and PCL diol 531 (10.5054 g, 19.78 mmol) were added into a 250 mL round flask with 80 mL of DMAc. The flask was immersed into an oil bath at 80 °C, and 19 drops of SnOct_2_ were added. The reaction mixture was stirred until the complete disappearance of the isocyanate groups following the NCO stretching vibration at 2270 cm^−1^ by FTIR. The PUPCL531 solution was cast into a leveled Teflon mold at 80 °C and the solvent was removed for 12 h. Finally, the obtained film was dried under vacuum for 24 h in order to assure the complete elimination of the residual solvent.

### 2.4. Preparation of the Polyurethane Based on PCL2054 with a 45% Hard Segment (PUPCL2054)

PCL-based polyurethane synthesized using PCL diol 2054 and 45% of hard segment, namely, PUPCL2054, was prepared by the prepolymer method. When the one-step method used for PUPCL531 was attempted for the PUPCL2054 synthesis, AMINE-OL did not dissolve completely during the reaction and for this reason the two-step procedure was used. Firstly, HDI (4.9423 g, 29.38 mmol) and PCL diol 2054 (11.0247 g, 5.37 mmol) were added into a 250 mL round flask with 39 mL of DMAc and 10 drops of SnOct_2_. The solution was stirred at 80 °C for 2 h. Then, the AMINE-OL (4.0638 g, 24.02 mmol) dissolved in 79 mL of DMAc was added and the temperature increased to 92 °C in order to achieve a better homogenization of the mixture. Later, 10 drops of SnOct_2_ were added and the reaction mixture was stirred until the complete disappearance of the isocyanate groups. The PUPCL2054 solution was cast into a leveled Teflon mold at 80 °C and the solvent was removed for 12 h. Finally, the obtained film was dried under vacuum for 24 h.

In both cases, the hard segment content was defined as [weight of HDI + weight of chain extender (AMINE-OL)/total weight of monomers × 100] as usually carried out in industry. The synthesis conditions and the most important properties of the synthetized PUs are reported in Table 1.

### 2.5. Characterization Methods

Nuclear magnetic resonance (^1^H NMR) spectra were recorded with a Varian Mercury 400 instrument (Palo Alto, CA, USA) using deuterated dimethylsulfoxide (DMSO-d_6_) as the solvent. The residual signal of the solvent (2.50 ppm) was used as a reference.

The thermal properties were investigated by differential scanning calorimetry (DSC), thermogravimetric analysis (TGA) and dynamic mechanical thermal analysis (DMTA). DSC analysis was performed in a Mettler Toledo 822e instrument (Mettler Toledo International Inc., Columbus, OH, USA) under nitrogen atmosphere (30 mL·min^−1^). Samples were sealed in aluminum pans and heated from 25 °C to 150 °C at 10 °C·min^−1^ in order to erase their thermal history. Then, a cooling scan to −90 °C at 10 °C·min^−1^ was performed, followed by a second heating scan to 150 °C at 10 °C·min^−1^. The glass transition temperature (*T_g_*) was taken as the midpoint of the transition from the second heating scan. 

TGA was performed in a Mettler Toledo TGA/SDTA 851^e^ instrument (Mettler Toledo International Inc., Columbus, OH, USA). Samples were heated from room temperature to 800 °C at 10 °C·min^−1^ under nitrogen atmosphere (30 mL·min^−1^).

DMTA tests of the samples were carried out using a DMA Q800 from TA Instrument (USA) in tension mode with an amplitude of 20 μm, a frequency of 1 Hz, a force track of 125% and a heating rate of 2 °C·min^−1^. Samples subjected to DMTA were cut from the casted films into rectangular specimens of approximately 20 mm × 5 mm × 0.70 mm.

Mechanical property characterization was carried out using an Instron, Universal Testing Machine (Model 3366, Instron, Norwood, MA, USA) at a strain rate of 10 mm·min^−1^ at room temperature. Measurements were performed on 3 specimens of approximately 35 mm × 8.5 mm × 0.70 mm with an initial length between the clamps of 20 mm. From these experiments, the Young’s modulus, the elongation at break and the tensile strength were obtained. 

Thermally activated shape-memory properties were studied using thermo-mechanical cycle experiments in a DMA Q800 TA Instrument (USA). First, the sample was heated above the corresponding transition temperature (*T_trans_*) and deformed to a set strain. The *T_trans_* used in this work was a temperature close to the UPy reversible network dissociation temperature (*T_dis_*), considering *T_trans_* < *T_dis_* [33]. Then, the material was cooled at the fixing temperature (*T_fix_*), maintaining the deformation in order to fix the temporary shape. Once the applied stress was removed, the recovery of the original shape occurred by re-heating the sample at the *T_trans_*. In particular, samples were heated at a *T_trans_* of 100 °C and 85 °C for PUPCL531 and PUPCL2054, respectively, for 5 min, and stretched at 50% of deformation by applying a constant deformation stress of 0.05 MPa·min^−1^. Then, they were quenched with a nitrogen flow at 25 °C under the same constant stress and the temporary shape was fixed after releasing the stress. The permanent shape was recovered upon heating (3 °C·min^−1^) to the corresponding *T_trans_*. Five different thermo-mechanical cycles were performed for each material. Strain fixity ratio (*R_f_*) and strain recovery ratio (*R_r_*) were calculated in order to quantify their shape-memory properties. In particular, *R_r_* is the ability to recover the original shape and was taken as the ratio of the recovered strain to the fixed strain, as given by the following equation:

(1) $$R_{r}\left(N\right)=\frac{\left(\varepsilon_{u}-\varepsilon_{p}\left(N\right)\right)}{\left[\varepsilon_{u}-\varepsilon_{p}\left(N-1\right)\right]}\times 100\%$$

*R_f_* is the ability to fix the temporary shape and it was taken as the ratio of the fixed strain to the deformed strain, as presented by Equation (2):

(2) $$R_{f}\left(N\right)=\frac{\varepsilon_{u}\left(N\right)}{\varepsilon_{m}}\times 100\%$$

where *ε_m_* is the deformed strain, *ε_u_* is the fixed strain, *ε_p_* is the recovered strain and *N* is the number of cycles.

## 3. Results and Discussion

**Figure 1:**
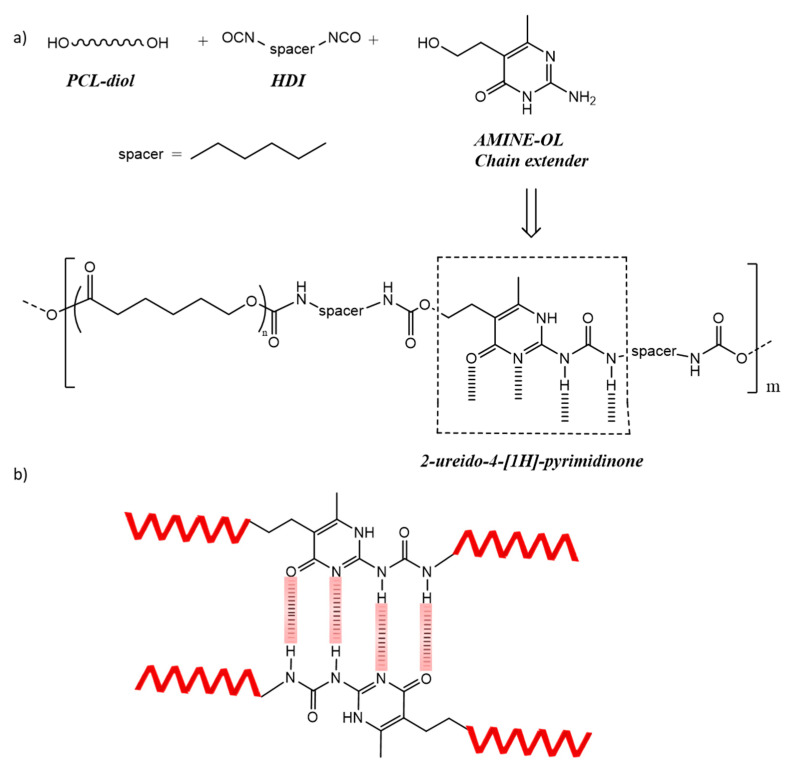
(**a**) Reaction scheme for PCL-based polyurethanes. (**b**) Schematic representation of the UPy network formation.

New 2-ureido-4-[1H]-pyrimidinone (UPy) self-complementary supramolecular PCL-based polyurethanes were successfully synthesized as shown schematically in Figure 1a. The UPy units are randomly distributed in the PU backbone; thus, the linear chains might be physically cross-linked as represented in Figure 1b.

**Figure 2:**
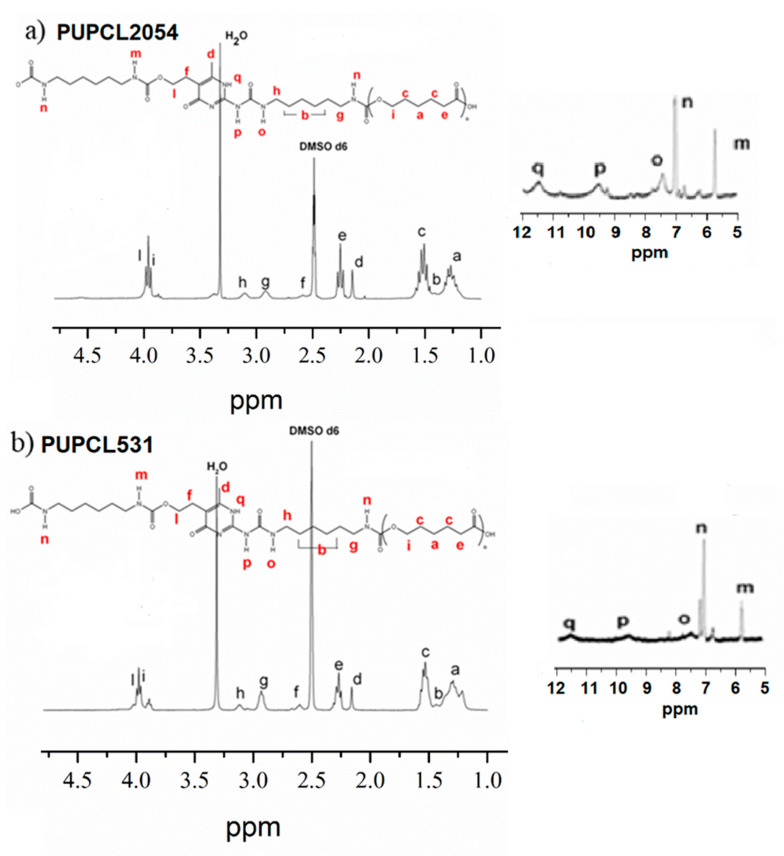
^1^H NMR spectra of (**a**) PUPCL2054 and (**b**) PUPCL531 in DMSO d_6_.

Two different PUs were synthetized using two PCL diols with different molecular weights, 531 and 2054 g·mol^−1^, respectively, and 45% of hard segment in both cases. The structures of both polyurethanes, based on PCL 2054 (PUPCL2054) and PCL 531 (PUPCL531) were confirmed by ^1^H NMR as reported in Figure 2a,b, respectively, indicating their successful formation.

For PUPCL2054, the characteristic peaks attributed to the UPy urea and urethane groups appeared at 11.53 ppm (δH^q^), 9.59 ppm (δH^p^), 7.47 ppm (δH^o^), 6.95 ppm (δH^n^) and 5.72 ppm (δH^m^). Meanwhile, the characteristic peaks of the methylene groups in the PCL backbone appeared at 3.87 ppm (δH^i^), 2.26 ppm (δH^e^), 1,52 ppm (δH^c^) and 1.20 ppm (δH^a^). Regarding PUCL531, the characteristic signals attributed to the UPy urea and urethane groups appeared at 11.55ppm (δH^q^), 9.54 ppm (δH^p^), 7.45 ppm (δH^o^), 7.00 (δH^n^) and 5.71 ppm (δH^q^). NMR measurements confirmed the presence of UPy N-H signals (signals q, p and o) [22].

**Figure 3:**
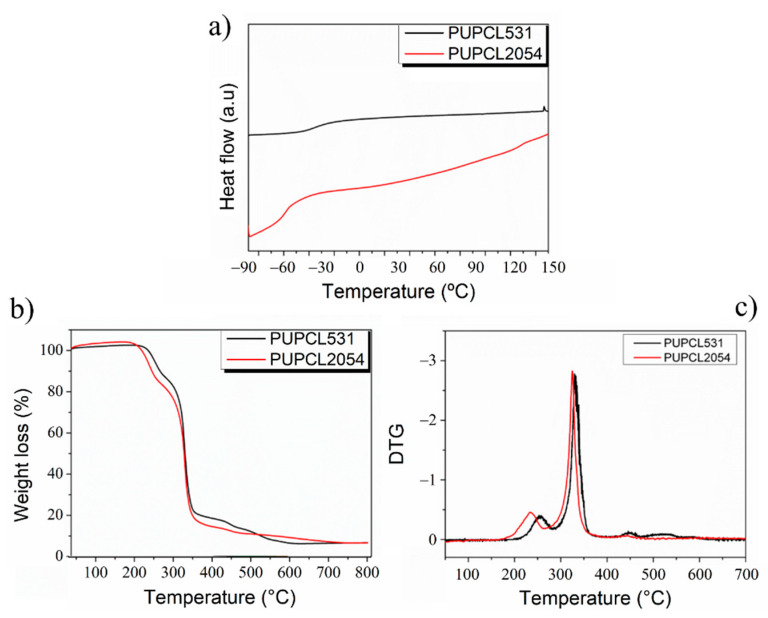
(**a**) DSC second heating scan of PUPCL531 (black) and PUPCL2054 (red). (**b**) TGA thermogram and (**c**) derivative of the weight loss in function of the temperature for both materials.

Thermal degradation and thermal transitions of the materials were investigated using TGA and DSC, respectively. In Figure 3, the thermograms obtained by DSC and TGA analysis for both PUPCL2054 and PUPCL531 are shown while the thermal results are reported in Table 1.

In particular, a single glass transition temperature was found at −38 °C and −68 °C for PUPCL531 and PUPCL2054, respectively, driven by the PCL diol. In fact, the *T_g_* shifted to lower temperatures as the chain length of the soft segment was increased, that is for PUPCL2054. This fact can be explained by considering that as the molecular weight of the PCL diol increases, the hindering effect to the polymeric chain motion of the hard segment (HS) is low and, accordingly, the glass transition temperature of the polymer occurs at a lower temperature, as previously demonstrated [34]. From the DSC analysis, we also found that the synthesized materials are amorphous and we expect that they are formed by a pure amorphous PCL phase and either an amorphous phase of PCL mixed with hard segments or a pure hard segment phase, with no detectable further thermal transition.

From the TGA thermogram, an initial decomposition was observed for PUPCL531, starting at 200 °C and finishing at 275 °C with a maximum loss rate at 250 °C and a total weight loss of 12%. This weight loss was attributed to the thermal decomposition of urethane and urea groups according to the literature [32,35]. The second decomposition step ranged from 280 °C to 360 °C with a maximum loss rate at 340 °C and a total weight loss of 68% and it was attributed to the thermal decomposition of PCL, as previously reported in the literature [36]. A low amount of carbonaceous residue was observed at 450 °C. PUPCL2054 showed a similar behavior. However, in this case, the thermal decomposition of the urethane and urea groups started earlier, around 180 °C, with a maximum loss weight at 215 °C, and finished at 260 °C with a total weight loss of 18%. Moreover, the thermal decomposition of the PCL chains started at around 265 °C and reached the maximum weight loss at 310 °C, corresponding to a total weight loss of 67%. Thus, increasing the SS length of the samples, the thermal degradation temperatures decreased, as it was previously reported by Petrović et al. [37] for polyurethanes with different soft segment lengths.

**Figure 4:**
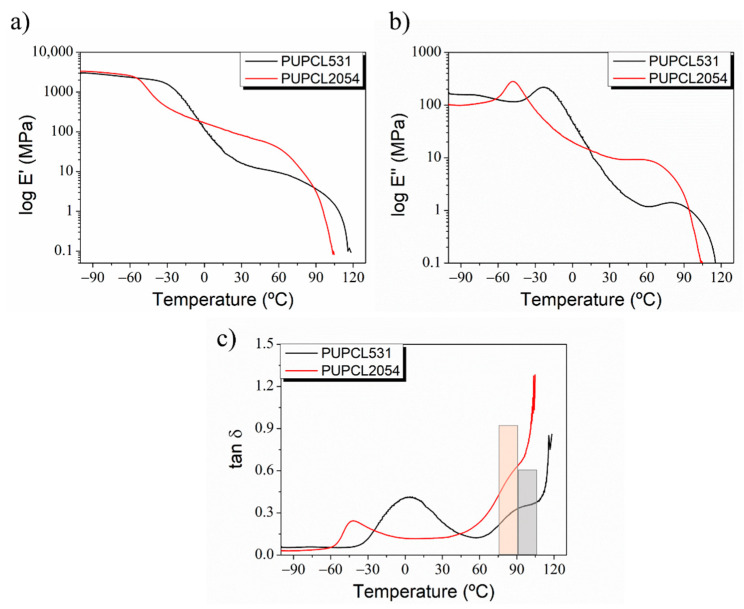
Dynamic mechanical thermal analysis: (**a**) storage modulus, (**b**) loss modulus and (**c**) tan δ for both samples.

The storage modulus (E’), loss modulus (E’’) and the damping factor (tan δ) as a function of temperature are reported in Figure 4.

The DMTA test confirmed the results obtained by DSC analysis. In fact, the *T_g_* of PUPCL2054 is located at a lower temperature than for PUPCL531, in fact, in the loss modulus curves (Figure 4b), PUPCL2054 showed a *T_g_* value (−47 °C) lower than that of PUPCL531(−23 °C). Therefore, after the drop in the *E*′ curve, ascribed to the *T_g_*, a rubber region was observed, from 25 °C to 70 °C for PUPCL531 and from −20 °C to 55 °C for PUPCL2054 (Figure 4a). Within this region, assumed to be an interphase of mixed PCL segments and hard segment, *E*′ decreased continuously and was interpreted as hydrogen bonds with different strengths being weakened at increasing temperatures. Due to the location of the UPy units in the main backbone, it is assumed that it is not very easy to order the UPy units to form quadrupole bonds. It has been demonstrated in simple urea bonds that initially the order in the hydrogen bonds is quite lousy and after thermal treatment at temperatures above the temperature own of the chain mobility, the urea bonds rearrange in more ordered hydrogen-bonded structures [38]. Therefore, in our case, UPy units could be hydrogen-bonded in different ways, less perfect than quadrupole bonds, not only among them but also with the urethane groups present in the polymer backbone. Thus, the collection of possible hydrogen bonds would relax at different temperatures, producing the descending plateau. As reported in the scientific literature, UPy motifs located in the lateral chains within the soft segment present more mobility, producing more ordered quadrupole bonds with higher transition temperatures [39]. At higher temperatures, a sharp decrease in the *E*′ and *E*′′ curves, which was related to the glass transition temperature of segregated hard segments, took place in both materials. Shoulders in the tan δ curves were observed around 100 °C and 85 °C for PUPCL531 and PUPCL2054, respectively. The DMTA results indicate that a supramolecular elastic network formed by H-bonds with gradual strength was obtained for both materials, the same result already reported in the scientific literature [40]. At low temperatures, H-bonds acted as non-covalent crosslinks and contributed to the high storage modulus. Above a maximum temperature, the samples did not allow the oscillatory frequency applied to reach the viscous flow state. This fact indicates that for the shape-memory test the samples have to be triggered under those maximum temperatures, considered as the *T_trans_* (highlighted areas in Figure 4c) where most of the hydrogen bonds within the interphase are weak, while the glass transition temperature of the hard segments has not yet been reached. These temperatures are 100 and 85 °C for PUPCL2054 and PUPCL531, respectively. Furthermore, PUPCL2054 showed higher *E*′ and *E*″ values in the rubbery region compared to PUPCL531, probably due to stronger UPy aggregations [41].

**Figure 5:**
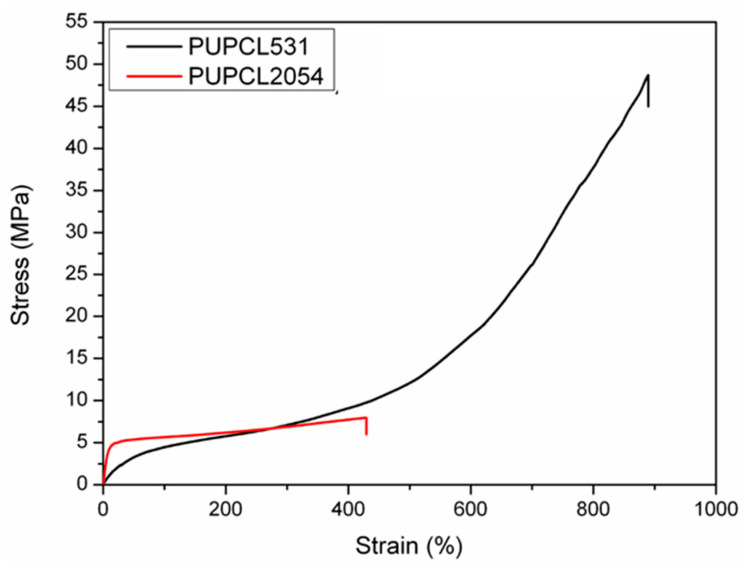
Stress–strain curves for PUPCL531 and PUPCL2054.

The mechanical properties of the samples were studied by tensile test and the stress–strain curves are showed in Figure 5.

Stress–strain curves showed the typical behavior of an elastomeric polymer for PUPCL531, with low values of elastic modulus and very high elongation at break. Tensile strength and elongation at break decreased with the increase in the SS length. Increasing the elongation, the orientation of the polymer chains is expected because of the rearrangement of the H-bonding until break. The results of the elastic modulus, tensile strength and elongation at break are reported in Table 2. Tensile test confirmed the DMTA results, showing that PUPCL2054 was able to form a more stable H-bonded network with stronger H-bonding. Indeed, when the sample was stretched, the material was found to be more rigid than PUPCL531, reaching a higher value of elastic modulus and a lower value of elongation at break. This behavior is probably due to the low mobility of the chains physically cross-linked by the H-bonds that when stretched are not able to rearrange.

**Figure 6:**
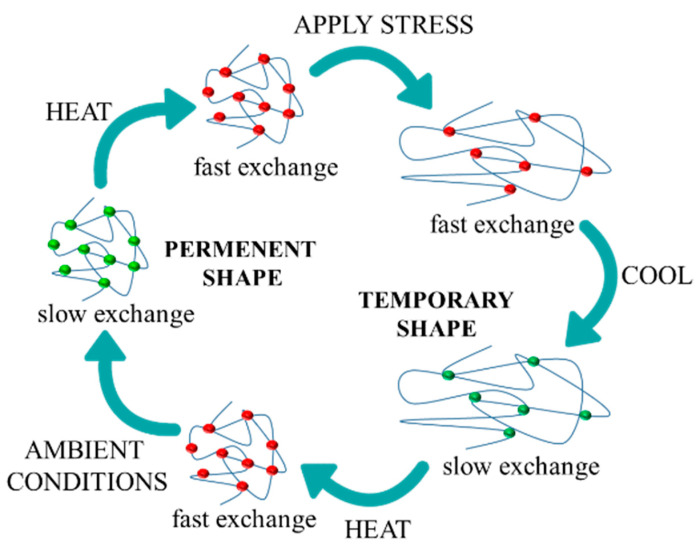
Schematic representation of shape-memory mechanism of our systems.

In the present work, the DMTA test was also used to investigate the shape-memory effects in both supramolecular polyurethanes, PUPCL531 and PUPCL2054. The proposed shape-memory mechanism assumes the “switching network”, the reversible non-covalent interactions based on the H-bonds of the UPy groups, which are capable of fixing the temporary shape. Moreover, the segregated hard segments of the PUs chains are proposed to be the “permanent network”. Figure 6 shows a schematic representation of the shape-memory mechanism. In particular, the sample was first equilibrated at *T_trans_* without any load, choosing as *T_trans_* a temperature of 100 and 85 °C for PUPCL2054 and PUPCL531, respectively.

**Figure 7:**
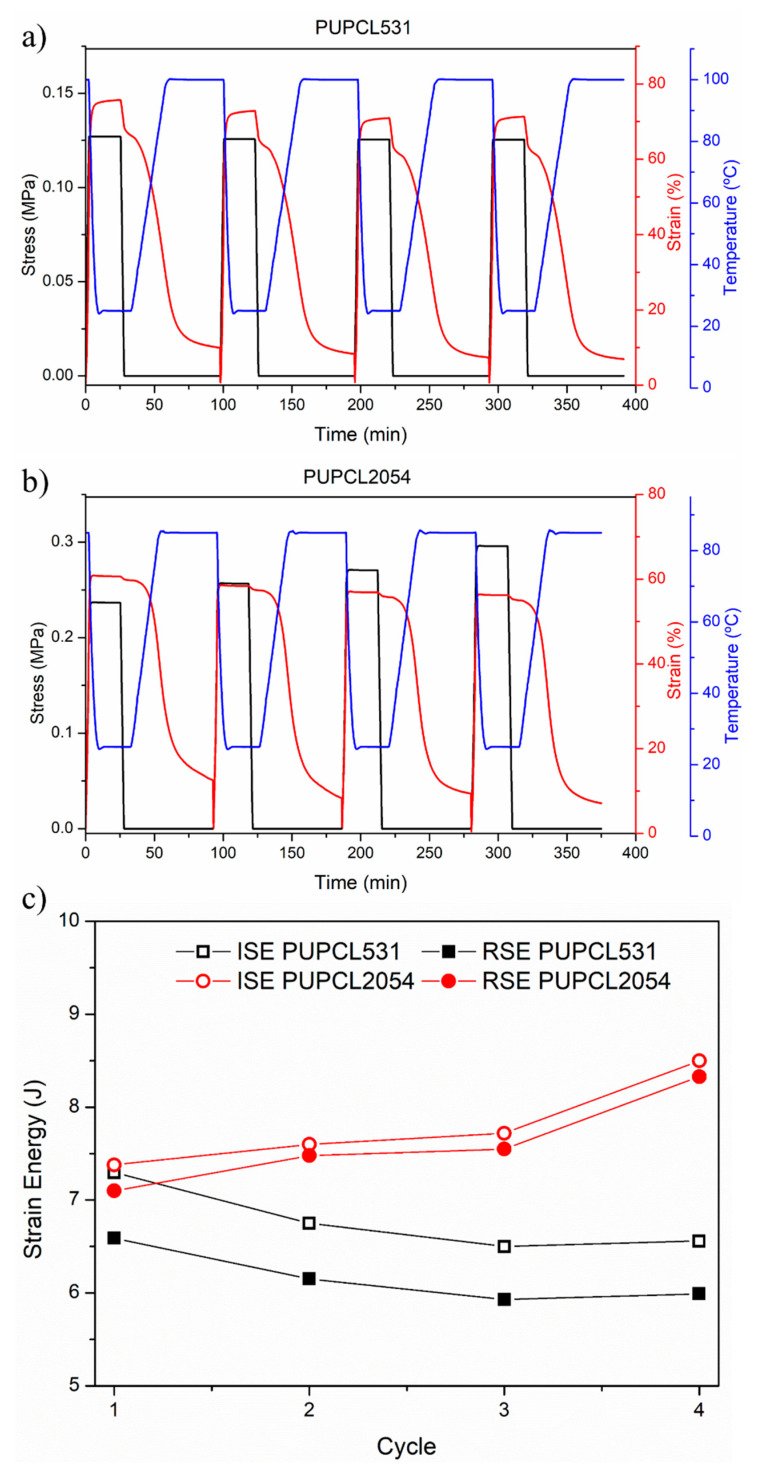
Thermo-mechanical cycles of the polyurethanes of (**a**) PUPCL531 and (**b**) PUPCL2054. (**c**) Strain energy comparison.

After 5 min at *T_trans_*, the sample was stretched at 50% of elongation, being the deformation at which the SME have been studied, *ε_m_*. Then, the temporary shape was fixed upon cooling to *T_fix_* at 25 °C for 15 min, removing the load, and the *T_trans_* was applied again, reaching the recovery of the original shape. Figure 7a,b present the evolution of strain, stress and temperature during the thermo-mechanical cycles for both PUPCL531 and PUPCL2054.

**Figure 8:**
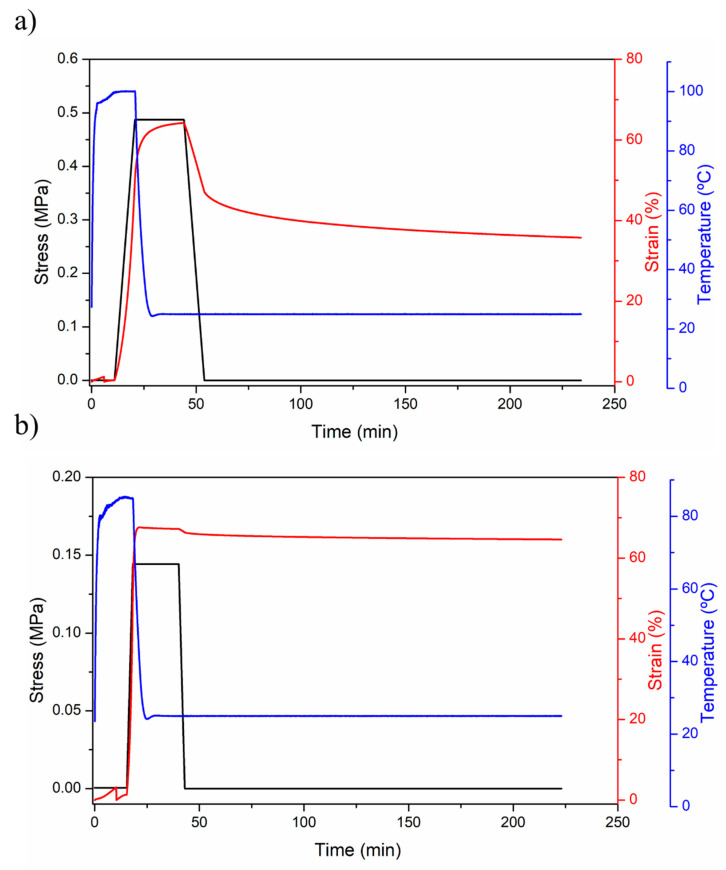
Two-dimensional stress–strain–temperature diagrams as a function of time for (**a**) PUPCL531 and (**b**) PUPCL2054.

It is easy to note that when the load was applied until 50%, both samples reached higher values of strain. This behavior may be attributed to the flexibility of the material at a high temperature of the permanent network that was not able to totally hold the applied stress. In order to avoid this lack of stiffness of the permanent network, Li et al. [18] synthetized a lightly cross-linked elastomeric UPy-functionalized PU, consequently preventing this phenomenon. Meanwhile, in our work, when the unloading was performed at *T_fix_*, the strain immediately decreased from *ε_m_* to *ε_u_*. Indeed, by removing the applied stress, stretched polymer chains can partially relax because they are now involved in a newly formed polymer network, consisting of H-bonds as net-points. Figure 8a,b show the stress–strain–temperature diagrams as a function of time for PUPCL531 and PU2054, respectively, where the ability of the material to maintain the fixed shape was studied at free-stress for 3 h.

For PUPCL531, by removing the load at *T_fix_*, the strain decreased from 64% to 47%, immediately losing 17% of strain. After 3 h at free-stress and *T_fix_*, the strain decreased from 47% to 36% (Figure 8a). Thus, it would be concluded that PUPCL531 was characterized by a fast strain relaxation and consequently a high dynamic network. Conversely, PUPCL2054 was able to hold the fixed temporary shape after 3 h (Figure 8b), probably due to the stronger UPy aggregations that compensate the high dynamicity of the network as obtained in the DMTA analysis. Indeed, at *T_fix_*, the strain relaxation is relatively slow. During the unloading, the strain decreased by only 2% and the sample recovered an additional 2% of strain after 3 h at free-stress. However, upon subsequent heating to *T_trans_*, the material quickly recovered, although not totally, its original shape. 

The obtained *R_r_* and *R_f_* values for all the cycles performed are shown in Table 3. Excellent strain fixity ratios were obtained in both supramolecular polyurethanes reaching values near 90% for PUPCL531 and close to 100% for PUPCL2054. The strain recovery ratio was increased for PUPCL531 from 85% to 89% in the last thermo-mechanical cycle. In the case of PUPCL2054, *R_r_* values increased during each cycle, starting from 79% and ending at 87% in the last thermo-mechanical cycle, also reaching excellent values in the case of *R_r_*. This fact indicates that both materials can memorize the original shape and can reversibly recover it from mechanical deformation.

It is important to underline that in these systems the materials were always above their soft segment glass transition temperature throughout the shape-memory cycles, showing good elasticity even in its temporary shape.

A qualitative study of the shape-memory behavior of the material was also carried out considering the strain energy of the material at different cycles. In Figure 7c, the real strain energy (RSE), obtained during the different thermo-mechanical cycles for both materials, is reported (filled symbols). In the same figure, the ideal strain energy (ISE), obtained by considering the PU as an ideal shape-memory material, is reported (unfilled symbols). It is known that a material with an ideal shape-memory behavior presents strain fixity equal to 100%, because *ε_u_* overlaps *ε_m_* and *ε_p_* is equal to 0, since in the ideal case the material is able to recover all the deformation [42]. Therefore, based on this hypothesis, we were able to calculate the ideal strain energy as the integral of the whole stress–strain curves obtained for the different cycles. Hence, considering that the material was not able to recover all the deformation, the real strain energy is calculated. Moreover, the ratio between the real and ideal strain energies allows us to calculate the energy efficiency for each cycle (Table 3). At the different cycles, the energy efficiency remains constant with values higher than 90% for PUPCL531, even after four cycles. In the case of PUPCL2054, the energy efficiency reaches values higher than 96%.

## 4. Conclusions

Two PCL-based supramolecular polyurethanes with UPy units capable of self-complementary quadruple hydrogen bonding and different lengths of SS were successfully synthetized. ^1^H NMR analyses confirmed the formation of the polyurethanes and the presence of the UPy unit in the polymer chains. DSC characterization revealed the totally amorphous morphology of the materials. Based on the DSC and DMTA results, it was concluded that both materials are characterized by a two-phase morphology. One formed by pure amorphous PCL phase and either an amorphous mixed phase of PCL and hard segments or a pure hard segment phase. The mechanism of their shape-memory behavior is based on the UPy as the switching domain and the network formed by the segregated hard segments as the permanent domain. The materials presented excellent shape-memory responses, reaching *R_f_* and *R_r_* values of around 87% and 89% for PUPCL531 and 98% and 87% for PUPCL2054, respectively. These materials are promising for applications where bio-based and potentially recyclable polymers with shape-memory properties are needed. 

## Figures and Tables

**Table 1:** Synthesis conditions, DSC and TGA results for PUPCL2054 and PUPCL531.

Sample	Reaction Path	Reaction T (°C)	PCL-Diol (g·Mol^−1^)	Hard Segment (%)	*T_g_* (°C)	*T*_5%_ (°C)	*T*_max1_ (°C)	*T_max_*_2_ (°C)
PUPCL531	One step	80	531	45	−38	200	230	330
PUPCL2054	Two steps	80/92	2054	45	−68	180	215	310

**Table 2:** Mechanical properties of the samples.

Sample	Elastic Modulus E (MPa)	Tensile Strength *σ_T_* (MPa)	Elongation at Break *ε* (%)
PUPCL531	13 ± 1	46 ± 5	880 ± 18
PUPCL2054	72 ± 3	6 ± 1	274 ± 4

**Table 3:** *R_r_* and *R_f_* values calculated for all the cycles of both materials. The energy efficiency values, defined as the ratio between the real and ideal values, are also summarized.

Sample	*R_r_* (%)				*R_f_* (%)				Energy Efficiency (%)			
Cycle Number	1	2	3	4	1	2	3	4	1	2	3	4
PUPCL531	85	87	88	89	87	87	87	86	90	91	91	91
PUPCL2054	79	85	83	87	98	98	98	98	96	98	98	98

## Data Availability

The data presented in this study are available on request from the corresponding authors.
